# A multicenter, real-world cohort study: effectiveness and safety of Azvudine in hospitalized COVID-19 patients with pre-existing diabetes

**DOI:** 10.3389/fendo.2025.1467303

**Published:** 2025-02-19

**Authors:** Yongjian Zhou, Zecheng Yang, Shixi Zhang, Donghua Zhang, Hong Luo, Di Zhu, Guangming Li, Mengzhao Yang, Xiaobo Hu, Guowu Qian, Guotao Li, Ling Wang, Silin Li, Zujiang Yu, Zhigang Ren

**Affiliations:** ^1^ Department of Infectious Diseases, State Key Laboratory of Antiviral Drugs, Pingyuan Laboratory, the First Affiliated Hospital of Zhengzhou University, Zhengzhou, China; ^2^ Department of Infectious Diseases, Shangqiu Municipal Hospital, Shangqiu, China; ^3^ Department of Infectious Diseases, Anyang City Fifth People’s Hospital, Anyang, China; ^4^ Guangshan County People’s Hospital, Xinyang, China; ^5^ Radiology Department, the First Affiliated Hospital of Zhengzhou University, Zhengzhou, China; ^6^ Department of Liver Disease, the Affiliated Infectious Disease Hospital of Zhengzhou University, Zhengzhou, China; ^7^ Department of Gastrointestinal Surgery, Nanyang Central Hospital, Nanyang, China; ^8^ Department of Infectious Diseases, Luoyang Central Hospital Affiliated of Zhengzhou University, Luoyang, China; ^9^ Department of Clinical Laboratory, Henan Provincial Chest Hospital Affiliated of Zhengzhou University, Zhengzhou, China; ^10^ Department of Respiratory and Critical Care Medicine, Fengqiu County People’s Hospital, Xinxiang, China

**Keywords:** COVID-19, Azvudine, diabetes, real-world, effectiveness, safety

## Abstract

**Introduction:**

During the Omicron infection wave, diabetic patients are susceptible to COVID-19, which is linked to a poor prognosis. However, research on the real-world effectiveness and safety of Azvudine, a common medication for COVID-19, is insufficient in those with pre-existing diabetes.

**Methods:**

In this retrospective study, we included 32,864 hospitalized COVID-19 patients from 9 hospitals in Henan Province. Diabetic patients were screened and divided into the Azvudine group and the control group, via 1:1 propensity score matching. The primary outcome was all-cause mortality, and the secondary outcome was composite disease progression. Laboratory abnormal results were used for safety evaluation.

**Results:**

A total of 1,417 patients receiving Azvudine and 1,417 patients receiving standard treatment were ultimately included. Kaplan−Meier curves suggested that all-cause mortality (P = 0.0026) was significantly lower in the Azvudine group than in the control group, but composite disease progression did not significantly differ (P = 0.1). Cox regression models revealed Azvudine treatment could reduce 26% risk of all-cause mortality (95% CI: 0.583-0.942, P = 0.015) versus controls, and not reduce the risk of composite disease progression (HR: 0.91, 95% CI: 0.750-1.109, P = 0.355). The results of subgroup analysis and three sensitivity analyses were consistent with the previous findings. Safety analysis revealed that the incidence rates of most adverse events were similar between the two groups.

**Conclusion:**

In this study, Azvudine demonstrated good efficacy in COVID-19 patients with diabetes, with a lower all-cause mortality rate. Additionally, the safety was favorable. This study may provide a new strategy for the antiviral management of COVID-19 patients with diabetes.

## Introduction

Coronavirus disease 2019 (COVID-19) emerged in December 2019, and severe acute respiratory syndrome coronavirus type 2 (SARS-CoV-2) infection has been endemic worldwide. The World Health Organization reported that by June 23, 2024, over 777 million cases of SARS-CoV-2 infection and 7.07 million deaths had been officially recorded globally (1). Owing to the high degree of transmission and lethality of SARS-CoV-2, COVID-19 continues to burden health care systems worldwide.

Diabetes is a major and enduring factor in the worldwide disease burden, with 537 million adults (aged 20-79 years) living with diabetes in 2021 globally. This figure is estimated to increase to 643 million by the year 2030 and 783 million by 2045 (2). Diabetes has been proven to be an independent risk factor for SARS-CoV-2 infection (3). Study also showed diabetes and hyperglycemia have been identified as predictors of worse clinical outcomes in COVID-19 patients (4–6). Data from China indicated that the COVID-19 case fatality rate was three times greater in patients with diabetes than in patients without diabetes (7). This difference may be related to the fact that people with diabetes are more susceptible to severe infections. However, effective strategies to alleviate the impact of COVID-19 are lacking for patients with diabetes (8). Although vaccination can reduce the effect of SARS-CoV-2 in high-risk populations, such as those with diabetes, it is ineffective in preventing infection caused by SARS-CoV-2 variants that have strong immune evasion capabilities (9, 10). Therefore, identifying efficient antiviral drugs for COVID-19 patients with preexisting diabetes is essential to reduce mortality and prevent disease progression.

Oral antivirals, including Molnupiravir, Nirmatrelvir−ritonavir (known as Paxlovid), and Azvudine, are recommended as priority therapeutic agents for patients with SARS-CoV-2 in China. Molnupiravir is the oral antiviral drug for mild-to-moderate COVID-19 with positive results of direct SARS-CoV-2 viral testing (11). Paxlovid is intended to treat mild to moderate COVID-19 patients who have high risk factors progressing to severe disease in adults (12). In China, Azvudine was officially approved as an alternative antiviral therapy for moderate COVID-19 patients in 2022 (13). Among these oral antivirals, Paxlovid and Molnupiravir have been well documented to be effective at decreasing hospitalization and death rates in patients infected with SARS-CoV-2 (14–17). According to previous studies, Azvudine can shorten the time of first nucleic acid negative conversion, reduce hospital stays, decrease the viral load, reduce hospital mortality and mitigate disease progression (18–20). Although a recent study revealed that Azvudine can reduce disease progression among hospitalized COVID-19 patients with pre-existing diseases (21), the efficacy and safety of Azvudine in the special high-risk population of patients with diabetes remain unknown. Therefore, the effectiveness and safety of Azvudine in COVID-19 patients with pre-existing diabetes warrant attention.

## Methods

### Study design and participants

This multicenter, retrospective cohort study included all hospitalized patients diagnosed with SARS-CoV-2 infection from nine hospitals in Henan Province, including the First Affiliated Hospital of Zhengzhou University, Henan Provincial Chest Hospital, Henan Infectious Disease Hospital, Luoyang Central Hospital, Nanyang Central Hospital, the Fifth People’s Hospital of Anyang, Shangqiu Municipal Hospital, Guangshan County People’s Hospital, and Fengqiu County People’s Hospital. The study period began on December 5, 2022, the first day that China officially announced the rolling back of strict anti-COVID-19 restrictions and ended on January 31, 2023. The study was approved by the Ethics Committee of the First Affiliated Hospital of Zhengzhou University (2023-KY-0865-001).

The patients included in the study were 1) hospitalized patients with a confirmed diagnosis of SARS-CoV-2 infection between December 5, 2022, and January 31, 2023; 2) patients receiving oral Azvudine or only standard treatment without any antiviral medication during their hospitalization; and 3) patients with preexisting diabetes. The exclusion criteria were 1) age less than 18 years, 2) receipt of antiviral medications other than Azvudine, and 3) pregnancy. The criteria for the diagnosis, treatment, and classification of SARS-CoV-2 infection can be found in the Diagnostic and Therapeutic Regimen for COVID-19 in China (Trial 10th Edition). Eligible patients receiving Azvudine treatment and standard treatment were divided into the Azvudine group and the control group, respectively.

### Data sources

Data for this research was collected from the digital medical files of patients at nine hospitals located in Henan Province. The information primarily consisted of demographic details, such as sex, age, body mass index (BMI), medical background, and clinical information such as admission date, diagnosis, treatment, laboratory tests, imaging results, ICU admission, and discharge or death date.

### Treatment exposure

During the study, patients who were admitted to the hospital with SARS-CoV-2 infection were given a daily dose of 5 mg of Azvudine orally as part of the treatment. Controls were selected from hospitalized patients with SARS-CoV-2 infection who received standard treatment including symptomatic treatment, monitoring of vital signs, oxygen therapy, administration of antimicrobial agents, management of comorbidities, immunotherapy, anticoagulation, and did not receive any antiviral agents during the observation period.

### Outcomes

All-cause mortality was the main outcome of the study, whereas composite disease progression was considered a secondary outcome. All-cause mortality was ascertained according to the electronic medical records. Disease progression was characterized by mortality, advancement to severe in patients with mild or moderate COVID-19.

Safety analysis was based on laboratory abnormalities in accordance with CTCAE 5.0, which classifies adverse events (AEs) into five grades: grade Grade 1 is mild, grade Grade 2 is moderate, grade Grade 3 is severe, grade Grade 4 is life-threatening, and Grade 5 is death related. Serious adverse events (SAEs) were defined as Grade ≥ 3 AEs that necessitated prompt medical intervention (22). Both any grades AEs and SAEs were assessed. The results were collected during the period from the administration of Azvudine to 5 half-lives after the last dose. In cases where multiple abnormalities were identified, the most severe outcome was selected for analysis.

### Baseline covariates

The baseline covariates of patients included age, sex, BMI, and severity of SARS-CoV-2 infection at diagnosis (mild cases were defined as only exhibiting representative symptoms of respiratory tract infection; moderate cases were defined as having a continuous high fever > 3 days but a respiratory rate < 30 breaths per minute or an oxygen saturation > 93%; severe cases were defined as having a shortness of breath with respiratory rate ≥ 30 breaths per minute, an oxygen saturation ≤ 93% at rest, a PaO2/FiO2 ≤ 300 mmHg, or lung infiltrates > 50%, or the need for mechanical ventilation, or shock, or ICU monitoring), concomitant hormone treatments at diagnosis, and comorbidities (hypertension, chronic liver diseases, cardio-cerebral diseases, chronic kidney diseases, and cancer). Furthermore, the key laboratory test parameters of patients upon diagnosis, such as high-density lipoprotein (HDL), low-density lipoprotein (LDL), cholesterol (CH), triglyceride (TG), alanine aminotransferase (ALT), aspartate aminotransferase (AST), alkaline phosphatase (ALP), gamma-glutamyl transpeptidase (GGT), albumin (ALB), creatinine (CREA), the glomerular filtration rate (e-GFR), C–reactive protein (CRP), procalcitonin (PCT), the prothrombin time (PT), activated partial thromboplastin time (APTT), total bilirubin (TBIL), neutrophils (Neut), lymphocytes (Lymph), and glucose (Glu), were collected.

### Statistical analysis

To adjust for the effects of confounders and baseline covariates (age, sex, disease severity, BMI, concurrent hormone therapy, hypertension, liver disease, cardiovascular disease, renal disease, primary malignancy, diabetes mellitus, hypertension, cardiovascular disease, and renal disease) on the evaluation of the intervention, we performed 1:1 PSM using a logistic regression model to identify the number of matched patients in the Azvudine and control groups and imputed missing data for baseline characteristics using multiple interpolation. After propensity score matching, baseline covariates were harmonized between the Azvudine and control groups. P > 0.05 combined with a standardized mean difference < 0.1 was considered balanced for the variables. We used the Kaplan−Meier method to estimate the overall cumulative hazard and statistically compared the Azvudine group with the control group using the log-rank sum test. To examine variables associated with the primary outcome, we used the Cox proportional hazards regression model to estimate hazard ratios (HRs) and 95% confidence intervals (95% CIs), with HRs greater than 1 suggesting a higher risk and HRs less than 1 indicating a lower risk. Schoenfeld residuals were used to evaluate the proportional risk hypothesis. To check for multicollinearity, we utilized the variance inflation factor (VIF), where a VIF greater than 5 suggests the existence of multicollinearity. The occurrence of adverse events was expressed as a proportion, and significant differences were calculated using the chi-square test.

To assess the strength of the calculations, we performed three sensitivity analyses. Initially, we tested the reliability of the results by interpolating missing values using means and then performing 1:1 PSM using a logistic regression model. Second, we used a probit model for 1:1 propensity matching for repeated testing. Finally, considering the time needed for blood concentrations of drugs to peak after drug administration, we limited the study population by excluding individuals who were discharged from the hospital on the initial day of drug administration or those who died. In addition, to explore possible differences in efficacy between patients with different characteristics, we performed subgroup analyses at each level of the above baseline covariates. All the statistical analyses were performed with R software (version 4.3.0, R Statistical Computing Foundation). Statistical significance was defined as a two-sided P < 0.05.

## Results

### Study population

**Figure 1:**
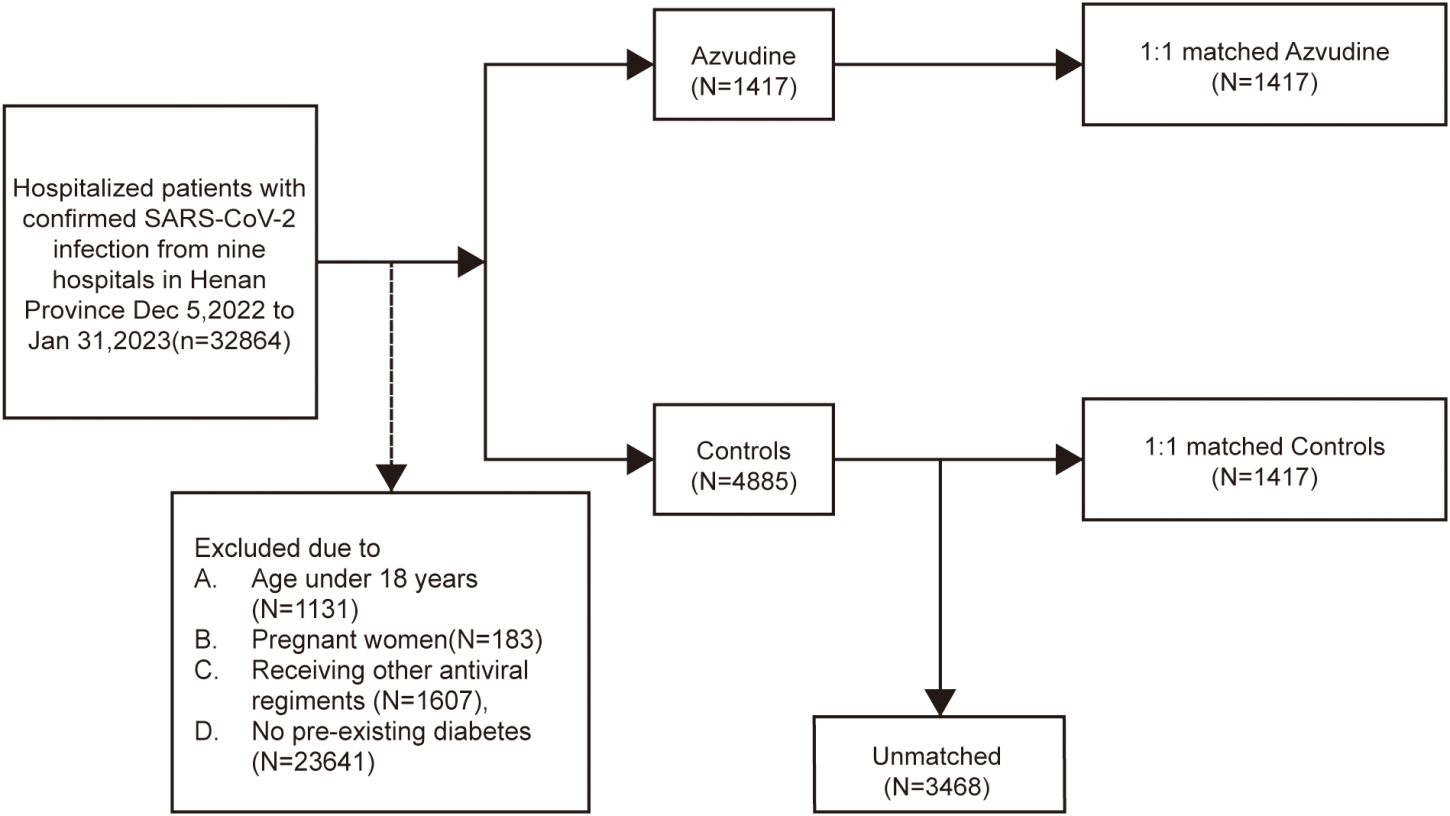
Cohort flow diagram. Study population flowchart showing the inclusion and exclusion of Azvudine recipients and their matched controls among hospitalized COVID-19 patients with pre-existing diabetes during the study period.

A total of 32,864 inpatients with SARS-CoV-2 infection were admitted to nine hospitals in Henan Province, China. According to the strict inclusion and exclusion criteria, 1417 patients who received Azvudine treatment and 4885 patients who did not receive any antiviral drugs were eligible for inclusion in the Azvudine group and control group, respectively. After PSM was used to balance the baseline characteristics of the two groups, a ratio of 1:1 was determined as the best matching ratio; thus, 1417 standard treatment patients were matched in the control group, and 1417 Azvudine recipients were matched in the Azvudine group (**Figure 1**, **Supplementary Table S1**).

The baseline characteristics of patients in the Azvudine and control groups before and after PSM are shown in **Table 1**. Before matching, the covariates significantly differed between groups, with male patients, older patients, more severe disease at diagnosis, hormone therapy, and hypertension being more prevalent in the Azvudine group than in the control group. Conversely, liver disease, cardiovascular disease and primary malignancies were less prevalent in the Azvudine group than in the control group. Furthermore, most laboratory tests also significantly differed between groups. After 1:1 PSM, the baseline characteristics of all covariates were balanced between the Azvudine and control groups, with p values greater than 0.05 (**Supplementary Figure S1**).

**Table 1:** Baseline characteristics of COVID-19 patients with pre-existing diabetes before and after PSM.

Characteristics	Before matching			After 1:1 matching		
	Control(n=4885)	Azvudine (n=1417)	P value	Control (n=1417)	Azvudine (n=1417)	P value
Age, mean (SD), year	68.03 (12.56)	70.18 (11.95)	<0.001	70.26 (12.28)	70.18 (11.95)	0.86
Gender n (%)			0.012			0.788
Male	2730 (55.9)	846 (59.7)		854 (60.3)	846 (59.7)	
Female	2155 (44.1)	571 (40.3)		563 (39.7)	571 (40.3)	
BMI, mean (SD), kg/m2	24.26 (3.73)	24.50 (3.85)	0.03	24.54 (3.72)	24.50 (3.85)	0.776
Severity at admission, n (%)			<0.001			0.494
Mild	494 (10.1)	78 (5.5)		92 (6.5)	78 (5.5)	
Moderate	3552 (72.7)	965 (68.1)		945 (66.7)	965 (68.1)	
Severe	839 (17.2)	374 (26.4)		380 (26.8)	374 (26.4)	
Concomitant systemic steroid, n (%)			<0.001			0.79
No	3826 (78.3)	818 (57.7)		826 (58.3)	818 (57.7)	
Yes	1059 (21.7)	599 (42.3)		591 (41.7)	599 (42.3)	
Hypertension n (%)			0.003			0.938
No	2005 (41.0)	519 (36.6)		522 (36.8)	519 (36.6)	
Yes	2880 (59.0)	898 (63.4)		895 (63.2)	898 (63.4)	
Hepatopathy n (%)			0.008			0.951
No	4246 (86.9)	1270 (89.6)		1268 (89.5)	1270 (89.6)	
Yes	639 (13.1)	147 (10.4)		149 (10.5)	147 (10.4)	
Cardio-cerebral diseases n (%)			<0.001			0.139
No	2385 (48.8)	821 (57.9)		781 (55.1)	821 (57.9)	
Yes	2500 (51.2)	596 (42.1)		636 (44.9)	596 (42.1)	
kidney diseases n (%)			<0.001			0.932
No	3916 (80.2)	1035 (73.0)		1038 (73.3)	1035 (73.0)	
Yes	969 (19.8)	382 (27.0)		379 (26.7)	382 (27.0)	
Primary malignant tumor n (%)			<0.001			0.72
No	4100 (83.9)	1315 (92.8)		1309 (92.4)	1315 (92.8)	
Yes	785 (16.1)	102 (7.2)		108 (7.6)	102 (7.2)	
Laboratory parameters mean (SD)						
Neutrophil, ×109/L	5.92 (4.11)	5.91 (3.91)	0.969	5.86 (4.00)	5.91 (3.91)	0.711
Lymphocyte, ×109/L	1.30 (7.37)	1.82 (28.56)	0.252	1.52 (13.61)	1.82 (28.56)	0.717
Glucose, mmol/L	9.87 (5.53)	10.33 (5.50)	0.006	10.33 (5.98)	10.33 (5.50)	0.988
Cholesterol, mmol/L	4.39 (5.29)	4.07 (2.20)	0.026	4.10 (2.35)	4.07 (2.20)	0.663
Triglyceride, mmol/L	2.05 (5.54)	1.67 (2.13)	0.011	1.81 (3.41)	1.67 (2.13)	0.176
High-density lipoprotein, mmol/L	1.05 (1.16)	1.08 (1.68)	0.582	1.10 (1.70)	1.08 (1.68)	0.649
Low-density lipoprotein, mmol/L	2.32 (1.66)	2.37 (2.35)	0.423	2.37 (2.26)	2.37 (2.35)	0.932
Alanine aminotransferase, IU/L	33.53 (124.70)	33.35 (73.09)	0.958	31.15 (68.07)	33.35 (73.09)	0.408
Aspartate aminotransferase, IU/L	41.90 (192.19)	39.54 (98.31)	0.656	33.75 (60.00)	39.54 (98.31)	0.059
Alkaline phosphatase, IU/L	91.16 (68.78)	80.07 (41.62)	<0.001	80.11 (38.23)	80.07 (41.62)	0.978
Gamma-glutamyl transpeptidase, IU/L	56.00 (99.41)	55.62 (95.13)	0.899	53.57 (92.37)	55.62 (95.13)	0.561
Albumin, g/L	38.58 (29.42)	37.55 (51.77)	0.339	38.87 (45.75)	37.55 (51.77)	0.472
Total bilirubin, μmol/L	12.90 (22.80)	11.43 (8.70)	0.018	11.15 (8.11)	11.43 (8.70)	0.379
Creatine, μmol/L	136.43 (271.09)	119.08 (208.44)	0.026	129.25 (174.79)	119.08 (208.44)	0.159
Glomerular filtration rate, ml/min	92.18 (121.24)	87.49 (110.61)	0.192	86.72 (105.23)	87.49 (110.61)	0.849
C-reactive protein, mg/L	53.14 (67.74)	57.62 (70.74)	0.03	57.50 (72.75)	57.62 (70.74)	0.963
Procalcitonin, ng/ml	2.51 (13.50)	1.79 (10.70)	0.064	1.90 (11.52)	1.79 (10.70)	0.79
Prothrombin time, s	14.44 (8.64)	17.11 (9.73)	<0.001	16.44 (11.87)	17.11 (9.73)	0.1
Activated partial thromboplastin time, s	29.03 (11.75)	26.57 (14.27)	<0.001	27.30 (10.30)	26.57 (14.27)	0.117

### All-cause mortality and composite disease progression

**Figure 2:**
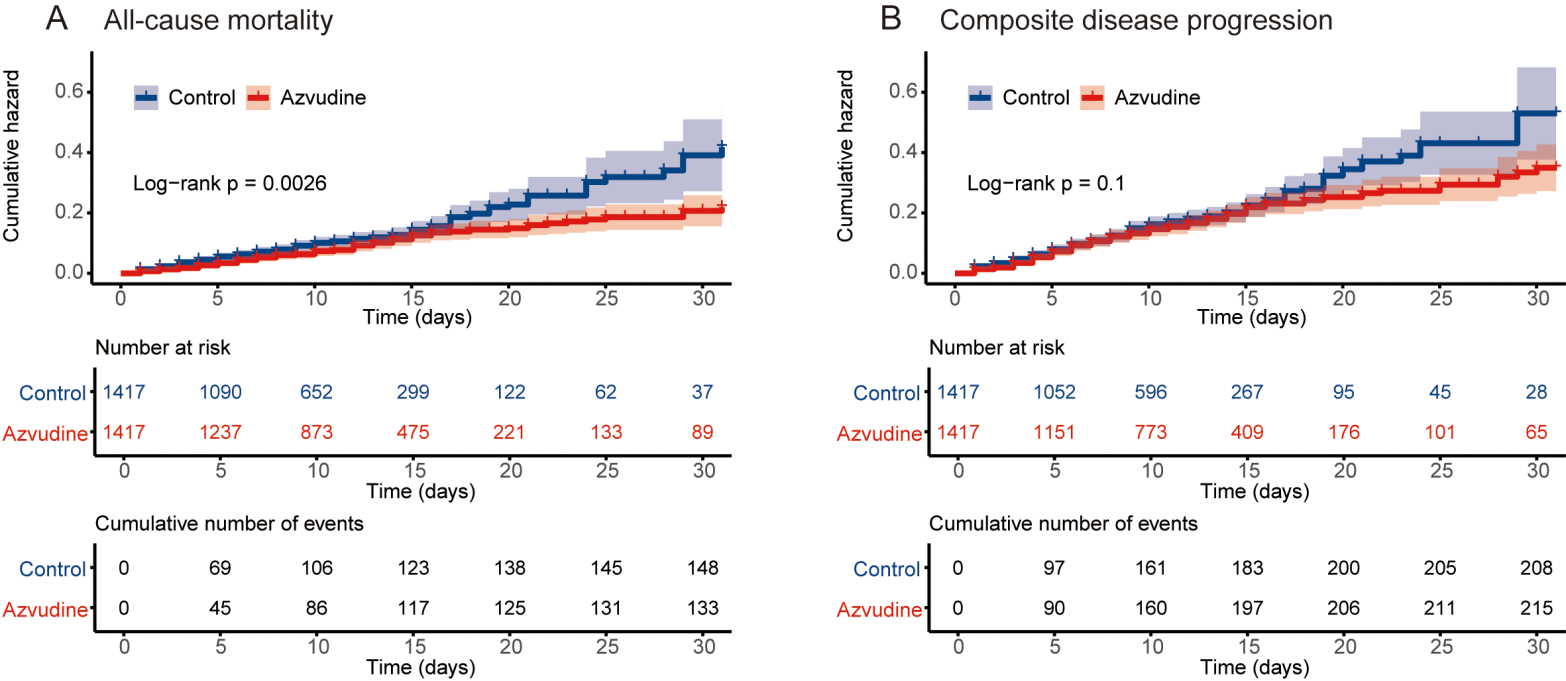
Kaplan–Meier curves of patients receiving Azvudine treatment versus controls. Cumulative hazard of all‐cause mortality **(A)** and composite disease progression **(B)**.

**Figure 3:**
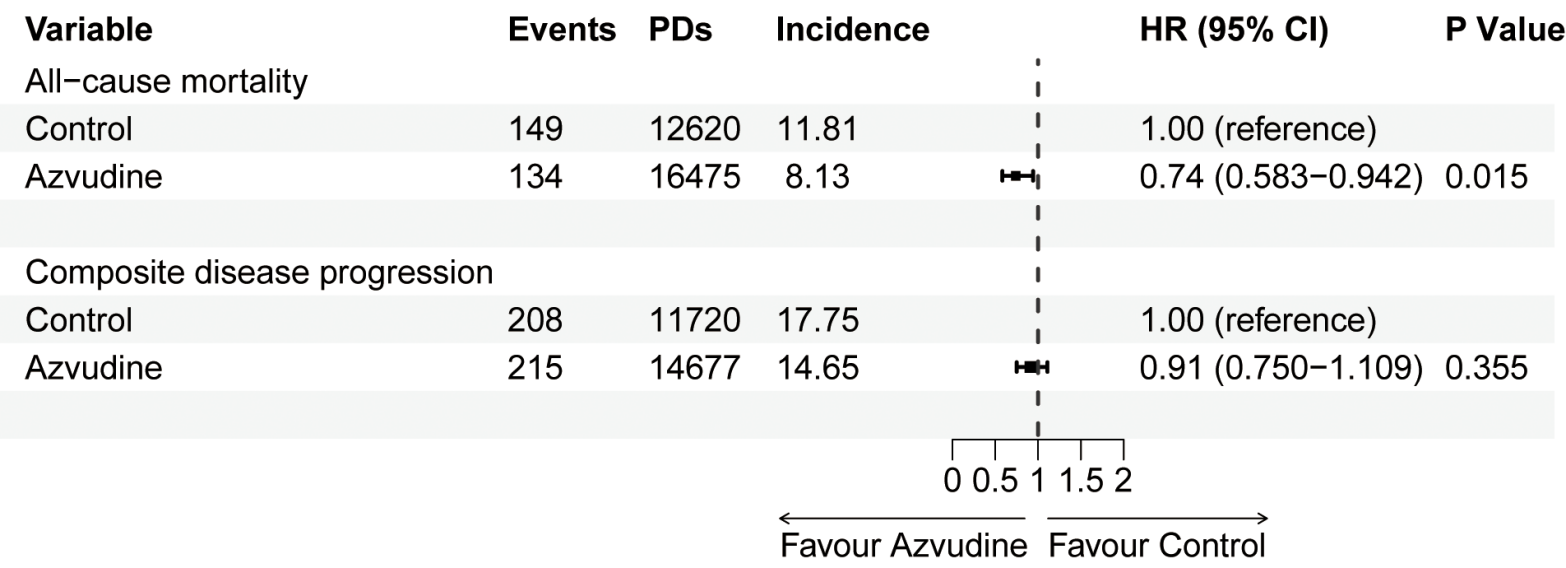
Multivariate Cox proportional hazards regression analysis of all-cause mortality and composite disease progression in patients receiving Azvudine and controls. HR, hazard ratio; 95% CI, 95% confidence interval. PDs, Person-days. Incidence: events/per 1000 PDs.

The main outcome was all-cause mortality. Among the 2834 patients included after PSM, 283 participants died, including 149 in the control group and 134 in the Azvudine group. Kaplan-Meier analysis revealed that, compared with the control treatment, Azvudine treatment significantly reduced the risk of all-cause mortality in patients (P = 0.0026) (**Figure 2A**). After multivariate adjustment via Cox regression analysis, the HR of all-cause mortality in the Azvudine group (P = 0.015) was 0.74 (95% CI: 0.583–0.942) compared with that in the control group (**Figure 3**).

Composite disease progression was the secondary outcome. During the follow-up period, 423 cases of compound disease progression occurred, including 208 cases in the control group and 215 cases in the Azvudine group. Kaplan-Meier analysis revealed no difference in composite disease progression between the Azvudine group and the control group (P = 0.1) (**Figure 2B**). The results of multivariate Cox regression analysis revealed that the HR for compound disease progression in the Azvudine group was 0.91 (95% CI: 0.750–1.109) (P = 0.355) (**Figure 3**).

The proportional hazards assumption was satisfied in the models for the primary and secondary outcomes. The VIF coefficients revealed that no multicollinearity existed (all VIFs < 5).

### Subgroup analysis

To further examine the association between antiviral therapy with Azvudine and all-cause mortality as well as composite disease progression across different groups, we stratified patients by sex, age, disease severity, the use of concomitant systemic steroids, therapies for diabetes, hemoglobin A1c (HbA1c) and the presence of multiple comorbidities. The subgroup analysis results showed no significant interaction between the different groups regarding the effect of Azvudine on reducing all-cause mortality, particularly in the diabetes therapies and HbA1c levels, which are critical for diabetic patients. To further assess the effect of various therapies for diabetes on the efficacy of Azvudine, considering the wide range of oral antidiabetic drugs and insulin, we selected the six most common treatment regimens and found that the efficacy of Azvudine varied among the groups, particularly in those using a combination of short-acting, rapid-acting, and long-acting insulin, where Azvudine exhibited a more pronounced protective effect on both all-cause mortality and composite disease progression. However, the subgroup analysis could only provide some indicative value due to potential bias from the limited data for certain treatments (**Supplementary Figure S8**). Overall, the results of the subgroup analysis suggested that Azvudine had a strong protective effect on reducing all-cause mortality in COVID-19 patients with diabetes mellitus (**Table 2**). Regarding composite disease progression, subgroup analysis revealed that Azvudine treatment did not adversely affect composite disease progression, and the results were consistent across subgroups (**Table 2**; **Supplementary Figure S8**).

**Table 2:** Subgroup analyses for the effectiveness of Azvudine in reducing the risk of all‐cause mortality and composite disease progression in COVID-19 patients with pre-existing diabetes.

Characteristics	All-cause mortality		Composite disease progression	
	HR (95%CI)	P value for interaction	HR (95%CI)	P value for interaction
Gender		0.193		0.197
Male	0.64 (0.48-0.84)		0.79 (0.63-1.00)	
Female	0.88 (0.56-1.36)		1.01 (0.71-1.42)	
Age		0.424		0.468
<=60 Year	0.88 (0.46-1.69)		0.72 (0.42-1.22)	
>60 Year	0.67 (0.52-0.86)		0.88 (0.71-1.08)	
Severity at admission		0.147		0.248
Mild	1.17 (0.40-3.40)		0.85 (0.27-2.65)	
Middle	1.02 (0.62-1.69)		1.27 (0.76-2.12)	
Severe	0.64 (0.48-0.84)		0.84 (0.68-1.04)	
Antidiabetic		0.659		0.592
No	0.67 (0.46−0.99)		0.81 (0.59−1.11)	
Oral antidiabetic drugs	0.54 (0.22−1.33)		0.64 (0.33−1.24)	
Insulin	0.67 (0.47−0.96)		0.83 (0.61−1.14)	
Combined use	0.97 (0.50−1.89)		1.09 (0.67−1.77)	
HbA1c		0.164		0.044
<=6%	3.52 (0.38−32.51)		3.70(1.00−13.76)	
>6%	0.72 (0.47−1.09)		0.89 (0.61−1.29)	
Unknown	0.64 (0.48−0.86)		0.78 (0.62−0.98)	
Concomitant systemic steroid		0.135		0.901
No	0.82 (0.59-1.14)		0.84 (0.65-1.09)	
Yes	0.58 (0.41-0.82)		0.87 (0.65-1.16)	
Hypertension		0.857		0.902
No	0.71 (0.49-1.04)		0.86 (0.63-1.18)	
Yes	0.69 (0.51-0.93)		0.85 (0.67-1.08)	
Hepatopathy		0.278		0.583
No	0.67 (0.52-0.86)		0.84 (0.68-1.03)	
Yes	0.94 (0.50-1.77)		0.97 (0.56-1.68)	
Cardio-cerebral diseases		0.651		0.576
No	0.77 (0.53-1.11)		0.92 (0.69-1.22)	
Yes	0.66 (0.49-0.89)		0.82 (0.63-1.06)	
Kidney diseases		0.687		0.466
No	0.68 (0.51-0.91)		0.82 (0.65-1.04)	
Yes	0.74 (0.50-1.10)		0.93 (0.67-1.29)	
Primary malignant tumor		0.987		0.448
No	0.70 (0.55-0.89)		0.84 (0.69-1.02)	
Yes	0.68 (0.24-1.96)		1.11 (0.50-2.49)	

### Sensitivity analysis

Three sensitivity analyses were conducted. First, we imputed missing values with the mean and performed Kaplan−Meier analysis (P = 0.0061) (**Supplementary Figure S2A**) and Cox regression analysis (HR: 0.69, 95% CI: 0.544-0.885, P = 0.003) on the processed data (**Supplementary Table S2**) (**Supplementary Figure S3**). Patients treated with Azvudine had a lower risk of all-cause mortality than control group patients receiving standard therapy. The risk of composite disease progression was consistent with the results of the previous dataset (Kaplan−Meier analysis, P = 0.32; Cox regression analysis, HR: 0.94, 95% CI: 0.772-1.150, P = 0.558) (**Supplementary Figures S2B**, **S3**).

Furthermore, using probit models for 1:1 PSM (**Supplementary Table S3**), both Kaplan−Meier analysis (P = 0.0036) (**Supplementary Figure S4A**) and Cox regression analysis (HR: 0.72, 95% CI: 0.564−0.913, P = 0.007) (**Supplementary Figure S5**) revealed that patients in the Azvudine group had lower all-cause mortality than patients in the control group. The composite disease progression rate was not different between the Azvudine group and the control group (Kaplan−Meier analysis, P = 0.29; Cox regression analysis, HR: 0.96, 95% CI: 0.786-1.170, P = 0.679) (**Supplementary Figures S4B**, **S5**).

Finally, repeated analysis after excluding patients discharged on the first day after admission revealed that the results remained robust (**Supplementary Table S4**): both Kaplan−Meier (P = 0.00035) (**Supplementary Figure S6A**) and Cox regression analyses (HR: 0.62, 95% CI: 0.482-0.793, P < 0.001) (**Supplementary Figure S7**) revealed that patients in the Azvudine group had lower all-cause mortality than those in the control group. For composite disease progression, the results of the Kaplan−Meier analysis (P = 0.089) were consistent with the original dataset (**Supplementary Figure S6B**), but the Cox regression analysis revealed (HR: 0.81, 95% CI: 0.655-0.992, P = 0.041) a reduction in the rate of composite disease progression in the Azvudine group compared with the control group (**Supplementary Figure S7**).

### Safety

Adverse events were recorded throughout the follow-up period for both the Azvudine and control groups, leading to a safety assessment being conducted (**Table 3**). The results demonstrated that during the follow-up period, most adverse events were not significantly different between the Azvudine and control groups. Compared with control subjects, Azvudine-treated subjects had a reduced risk of developing elevated creatinine (16% in the Azvudine group vs. 22% in the control group, P = 0.021). Among those with Grade ≥ 3 adverse events, patients in the Azvudine group were more likely to experience increased GGT levels (1.7% in the Azvudine group vs. 0% in the control group, P = 0.041). In summary, the findings indicate that the safety of Azvudine is relatively favorable, with a low incidence of adverse events.

**Table 3:** Incidence of adverse events in the study population receiving standard or Azvudine treatment.

Adverse events (n, %)	Available data^a^		All grades			Grade ≥ 3^b^		
	Control	Azvudine	Control	Azvudine	P value	Control	Azvudine	P value
Lymphocyte count decreased	567	833	123 (22%)	179 (21%)	>0.9	74 (13%)	109 (13%)	>0.9
Lymphocyte count increased	567	833	3 (0.5%)	6 (0.7%)	0.7	2 (0.4%)	0 (0%)	0.2
Neutrophil count increased	144	191	12 (8.3%)	12 (6.3%)	0.5	3 (2.1%)	1 (0.5%)	0.3
PLT count decreased	401	578	50 (12%)	76 (13%)	0.8	16 (4.0%)	24 (4.2%)	0.9
Anemia	338	402	85 (25%)	126 (31%)	0.063	24 (7.1%)	38 (9.5%)	0.2
Hypoglycemia	278	379	5 (1.8%)	6 (1.6%)	>0.9	2 (0.7%)	2 (0.5%)	>0.9
Hypercholesterolemia	61	119	4 (6.6%)	4 (3.4%)	0.4	0 (0%)	0 (0%)	
Hypertriglyceridemia	40	72	7 (18%)	13 (18%)	>0.9	0 (0%)	2 (2.8%)	0.5
ALT increased	459	721	68 (15%)	104 (14%)	0.9	10 (1.4%)	10 (1.4%)	0.3
AST increased	485	757	26 (5.4%)	29 (3.8%)	0.2	17 (3.5%)	14 (1.8%)	0.068
ALP increased	348	534	3 (0.9%)	2 (0.4%)	0.4	1 (0.3%)	0 (0%)	0.4
GGT increased	263	356	26 (9.9%)	51 (14%)	0.1	0 (0%)	6 (1.7%)	0.041
Hypophosphatemia	201	279	47 (23%)	48 (17%)	0.094	0 (0%)	0 (0%)	
Hypokalemia	551	793	122 (22%)	188 (24%)	0.5	30 (5.4%)	62 (7.8%)	0.09
Hyperkalemia	551	793	26 (4.7%)	32 (4.0%)	0.5	5 (0.9%)	7 (0.9%)	>0.9
Hyperuricemia	415	608	27 (6.5%)	29 (4.8%)	0.2	0 (0%)	0 (0%)	
CREA increased	379	589	82 (22%)	93 (16%)	0.021	1 (0.3%)	7 (1.2%)	0.2

ALT, alanine aminotransferase; AST, aspartate aminotransferase; ALP, alkaline phosphatase; GGT Glutamyltransferase; PLT, platelets; CREA, creatinine.

^a^Number of people who completed follow-up data collection for this indicator.

^b^Severity grades were defined according to the National Cancer Institute Common Terminology Criteria for Adverse Events (CTCAE), version 5.0.

## Discussion

The COVID-19 epidemic poses serious risks for diabetes patients worldwide. Studies have shown that diabetes is highly prevalent among hospitalized patients diagnosed with COVID-19 (23–25). A systematic review and meta-analysis indicates that among the 29,874,938 COVID-19 infected patients included from 60 countries and regions, the pooled prevalence of diabetes was 14.7%(95%CI: 12.5-16.9) (26). Studies have demonstrated that COVID-19 patients with pre-existing diabetes tend to have negative outcomes linked to high blood glucose levels. An observational study from the United States suggests that patients with diabetes or uncontrolled hyperglycemia have longer hospital stays (5.7 vs 4.3 days, P < 0.001) and significantly higher mortality rates than patients without diabetes or uncontrolled hyperglycemia (28.8% vs 6.2% P < 0.001) (27). A real-world study from China indicates that COVID-19 patients with pre-existing Type 2 Diabetes have a poor prognosis compared to those with well-controlled blood sugar. They require more medical interventions and are at a higher risk of multi-organ damage and death (28).Therefore, we need to find effective drugs that can prevent or treat SARS-CoV-2 infection in diabetic patients.

Azvudine, the first oral anti-SARS-CoV-2 drug in China, has been designated a priority for treating patients infected with SARS-CoV-2 (29). A retrospective cohort study showed that Azvudine can reduce the in-hospital mortality of COVID-19 patients in overall population (OR:0.375, 95% CI:0.225–0.623, *P <* 0.001) (30). A single-center, real-world experience demonstrated that using Azvudine was linked to a lower risk of all-cause mortality than without using Azvudine in COVID-19 patients with pre-existing cardiovascular diseases (HR:0.431, 95% CI:0.252-0.738, *P =* 0.002) (31). However, the safety and efficacy of Azvudine in COVID-19 patients with pre-existing diabetes remains unknown. To assess the safety and efficacy of Azvudine in the prevention and treatment of COVID-19 patients with pre-existing diabetes, a multicenter, large cohort study was performed. A total of 2834 participants were enrolled. A total of 1417 of them were treated with Azvudine, and the remaining patients received standard care as a control. To the best of our knowledge, this is one of the largest multicenter real-world studies on hospitalized COVID-19 patients with pre-existing diabetes. Among hospitalized diabetic patients infected with SARS-CoV-2, all-cause mortality was significantly lower in patients treated with Azvudine. However, the study revealed that treatment with Azvudine did not lead to a substantial decrease in composite disease progression. The same results were observed in various subgroup analyses. These results provide strong evidence for the efficacy of Azvudine in the treatment of diabetic patients infected with SARS-CoV-2.

To the best of our knowledge, the evidence from some previous retrospective studies examining Azvudine treatment in the COVID-19 cohort is not sufficient because only one method of analysis and subgroup analysis was used (21). To validate the reliability of the results, three sensitivity analyses were performed in this study to iteratively confirm the robustness of the results. First, to reduce the effect of missing value imputation, we reanalyzed the data via mean imputation instead of multiple imputation. Second, we used Poisson regression instead of logistic regression in the PSM to control confounding factors. Third, because the drug takes time to take effect, we excluded some patients who were discharged because of improvement, death, or disease progression on the day they received Azvudine and reanalyzed the data. All three methods of sensitivity analysis confirmed the results of this study, reaffirming the reliability of our findings. Most previous studies have ignored the effects of laboratory indicators as confounders on outcomes (21), but some studies have indicated a link between routine blood tests and mortality in hospitalized COVID-19 patients (32, 33), suggesting that many of our laboratory test indicators may also be potential confounders. Therefore, in this study, we rigorously controlled for confounders, such as demographic characteristics, comorbidities, medication use, disease severity, and laboratory indicators.

To verify the safety of Azvudine in the treatment of diabetic patients infected with SARS-CoV-2, various indicators of adverse events, including electrolytes, hepatic and renal function, and blood counts from patients’ electronic medical records, were included in the study, and we comprehensively assessed the various adverse events that occurred. The results revealed that only a few adverse events occurred in Azvudine treated patients compared with those in patients receiving standard therapy, but the overall number of adverse events was within acceptable limits.

Diabetes and COVID-19 represent a bidirectional relationship. Hyperglycemia can lead to poor outcomes in COVID-19 through various mechanisms. The expression of ACE2 receptors is upregulated in patients with pre-existing diabetes, making diabetic patients more susceptible to SARS-CoV-2 infection (34). Hyperglycemia can also affect the glycolytic process, leading to increased production of mitochondrial reactive oxygen species and activation of hypoxia-inducible factor 1α. It will promote the replication of SARS-CoV-2 in monocytes, exacerbating infection (35). At the same time, COVID-19 can activate the immune system, leading to the production of a range of cytokines such as interleukin-6 (IL-6) and tumor necrosis factor-alpha (TNF-α), which induce insulin resistance and hyperglycemia (36). Therefore COVID-19 Patients with pre-existing diabetes not only require glucose lowing therapies to prevent hyperglycemia, but also need treatment to fight the infection and inflammation. However, standard treatment such as using hormones may lead to the exacerbation of hyperglycemia (37). In addition, diabetic patients often have many comorbidities, including microvascular disease and cardiovascular disease. Ineffective control of viral infections greatly increases the difficulty of treatment. Therefore, Azvudine may aid the treatment of diabetic COVID-19 patients with complicated conditions and poor blood sugar control. Azvudine is absorbed by the human body and accumulates in the thymus in an active form, which can effectively inhibit the replication of SARS-CoV-2 and protect thymic immune function to treat COVID-19 (20). In this study, Azvudine effectively reduced all-cause mortality in patients of all ages, sexes and comorbidities. It did not worsen composite disease progression. In addition, Azvudine is scheduled to be added to the National Health care Institutes’ reimbursement list in July 2022, making it a more cost-effective option than other antiviral therapies while maintaining its safety and efficacy (38).

Our study has several shortcomings. First, vaccination status was not included in the baseline characteristics, but given the high vaccination rate in China, the lack of this information may not have had a large effect on the results. Second, we did not consider time to nucleic acid turnaround as a treatment effect to be evaluated because testing for SARS-CoV-2 was no longer essential during the later phases of the outbreak. Third, insulin use has been linked to unfavorable outcomes in COVID-19 patients (39). However, this factor was not controlled for in this study. Fourth, the majority of participants in this study were infected with the omicron strain, and whether Azvudine is effective against other variants remains unknown.

## Conclusion

In conclusion, findings from this extensive retrospective cohort study conducted across multiple centers indicate that Azvudine treatment in hospitalized COVID-19 patients with preexisting diabetes significantly reduces all-cause mortality and does not worsen composite disease progression, despite a very low number of adverse events that are within the range of expectation. These positive results provide valuable support for the use of Azvudine in clinical practice.

## Data Availability

The original contributions presented in the study are included in the article/**Supplementary Material**. Further inquiries can be directed to the corresponding authors.
