# Supplementary material for: A multicenter, real-world cohort study: effectiveness and safety of Azvudine in hospitalized COVID-19 patients with pre-existing diabetes
Source: Front Endocrinol (Lausanne). 2025 Feb 19;16:1467303. doi: 10.3389/fendo.2025.1467303 (PMC11879813; doi:10.3389/fendo.2025.1467303)
Supplement: Supplementary file 1 [file DataSheet1.docx]

**Supplementary Figures legends**

**
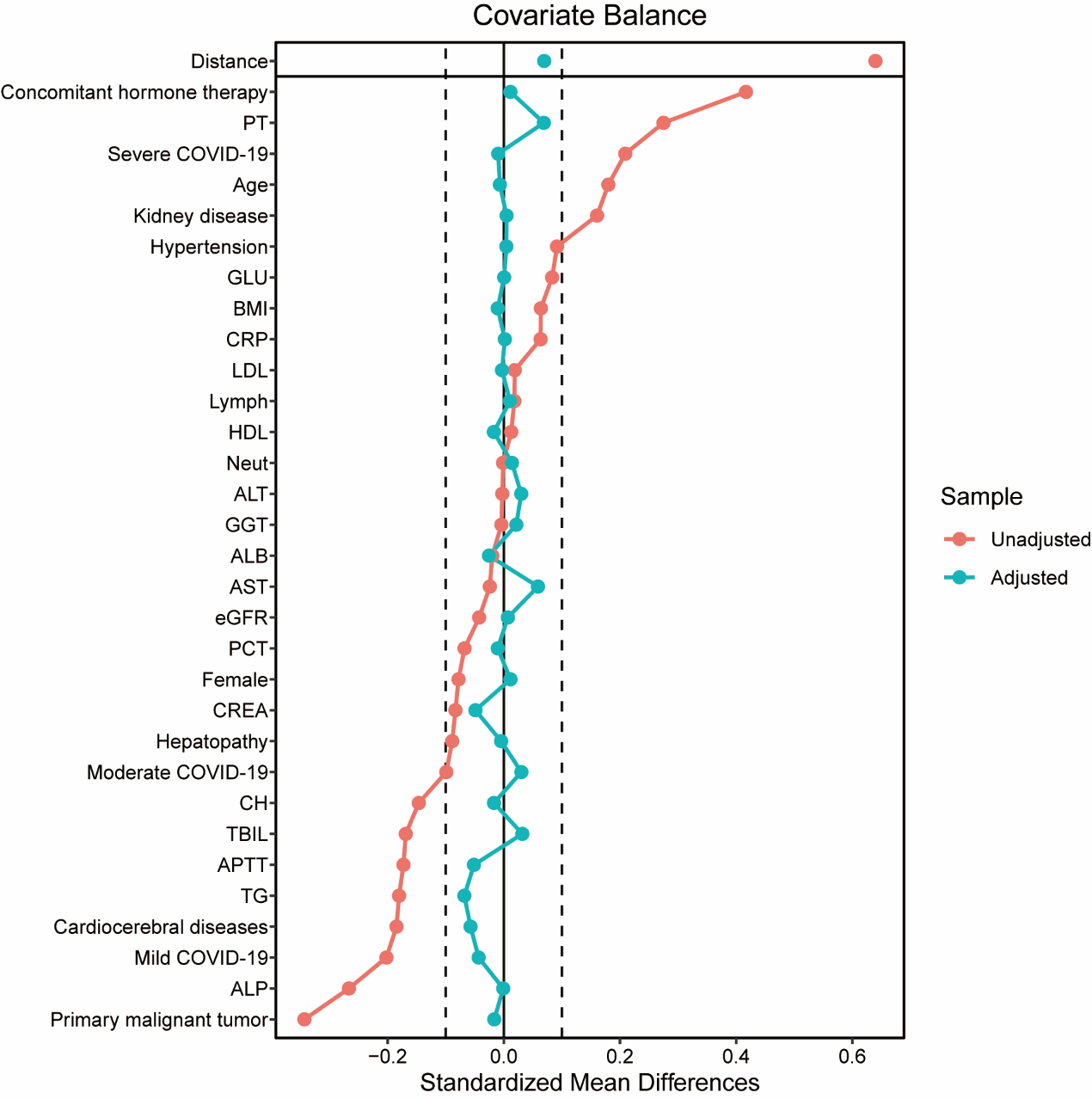
**

**Figure S1 Baseline characteristics before and after propensity score matching**

GFR, glomerular filtration rate; Neut, neutrophils; ALT, alanine aminotransferase; APTT, activated partial thromboplastin time; PCT, procalcitonin; GGT, gamma-glutamyl transpeptidase; ALP, alkaline phosphatase; AST, aspartate aminotransferase; Lymph, lymphocytes; LDL, low-density lipoprotein; TBIL, total bilirubin; HDL, high-density lipoprotein; CRP, C–reactive protein; CH, alanine cholesterol; Glu, glucose; PT, prothrombin time; TG, triglyceride; CREA, creatinine; ALB, albumin.


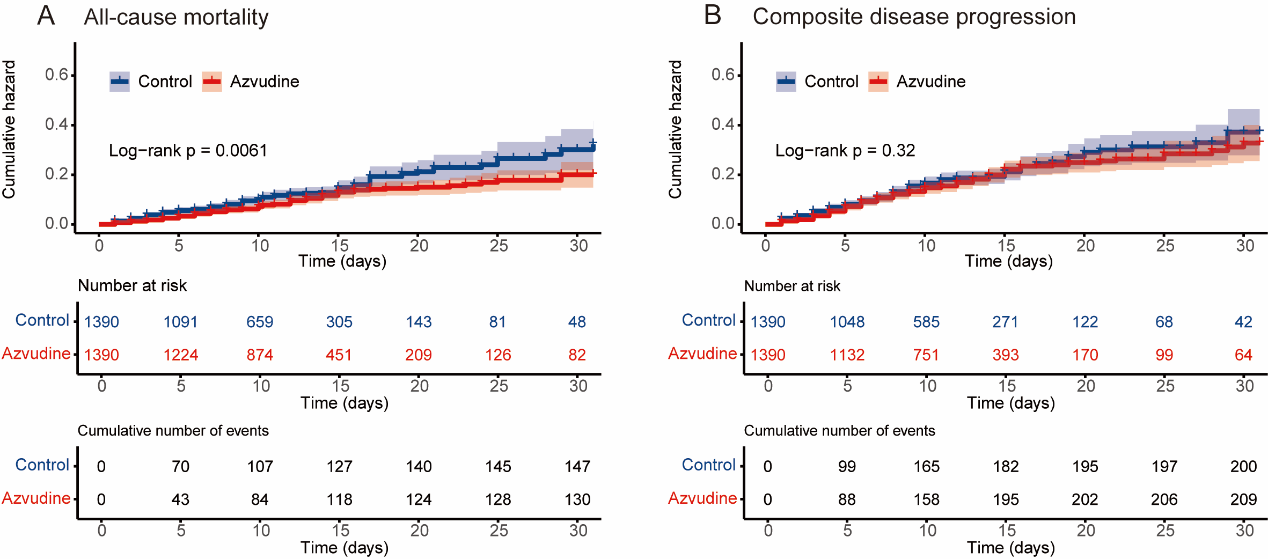


**Figure S2 Kaplan–Meier curves of patients receiving Azvudine treatment versus control in the sensitivity analysis where the missing data was filled up with the mean value.** Cumulative hazard of all‐cause mortality (A) and composite disease progression (B).


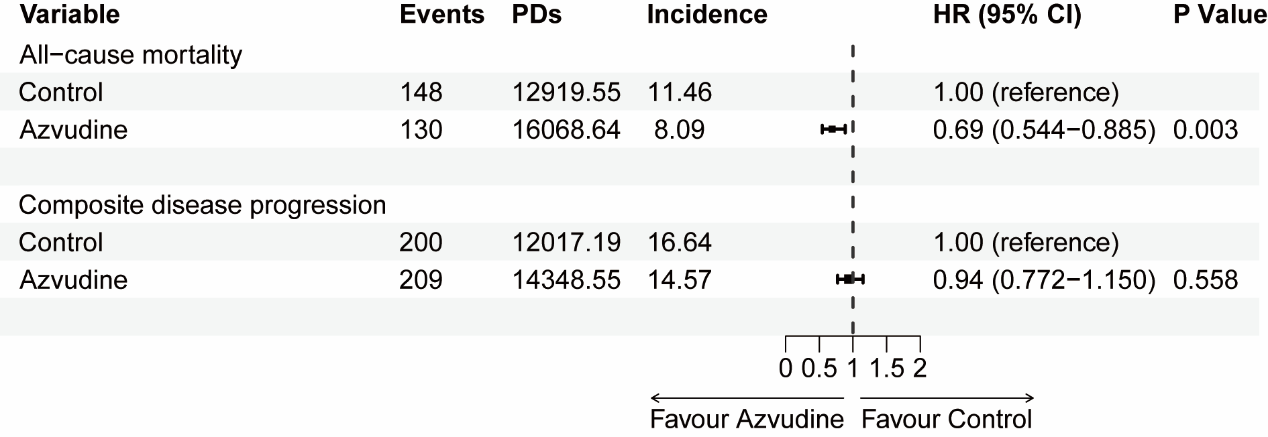


**Figure S3 Multivariate Cox proportional hazards regression analysis of all-cause mortality and composite disease progression in patients receiving control and Azvudine treatment in the sensitivity analysis where the missing data was filled up with the mean value.** Adjusted for all baseline covariates in Table S2. HR: Hazard Ratio; 95% CI: 95% confidence interval. PDs: Person-days. Incidence: events/per 1000 PDs.


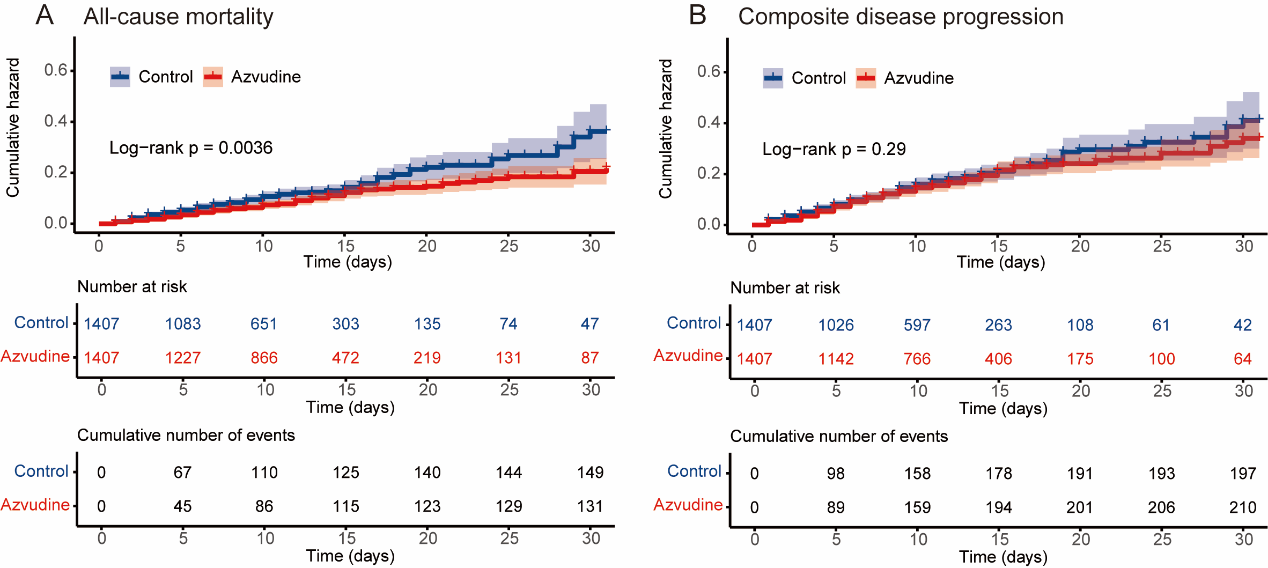


**Figure S4 Kaplan–Meier curves of patients receiving Azvudine treatment versus control in the sensitivity analysis where propensity score matching was performed using probit method.** Cumulative hazard of all‐cause mortality (A) and composite disease progression (B).


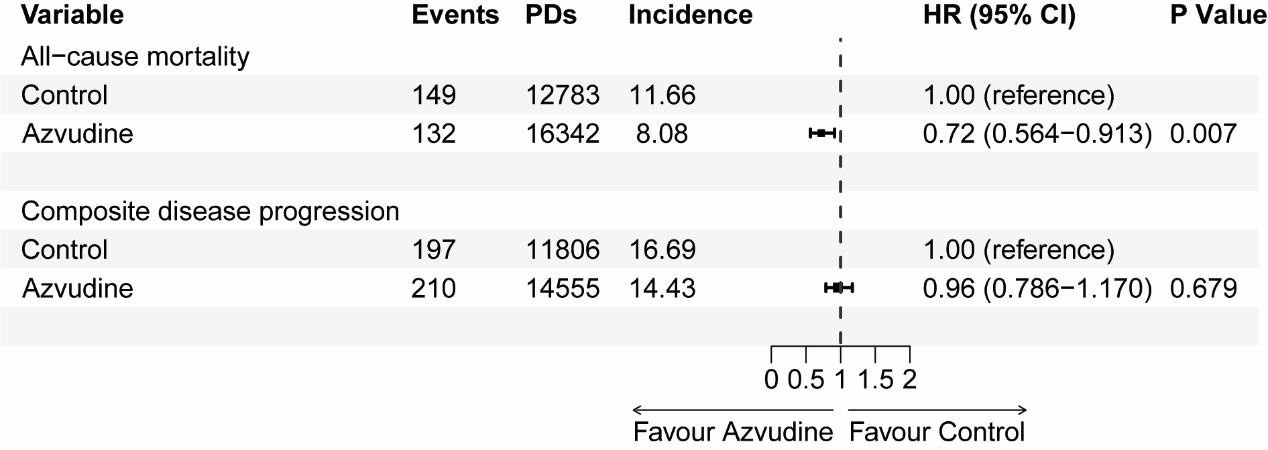


**Figure S5 Multivariate Cox proportional hazards regression analysis of all-cause mortality and composite disease progression in patients receiving control and Azvudine treatment in the sensitivity analysis where propensity score matching was performed using probit method.** Adjusted for all baseline covariates in Table S3. HR: Hazard Ratio; 95% CI: 95% confidence interval. PDs: Person-days. Incidence: events/per 1000 PDs.


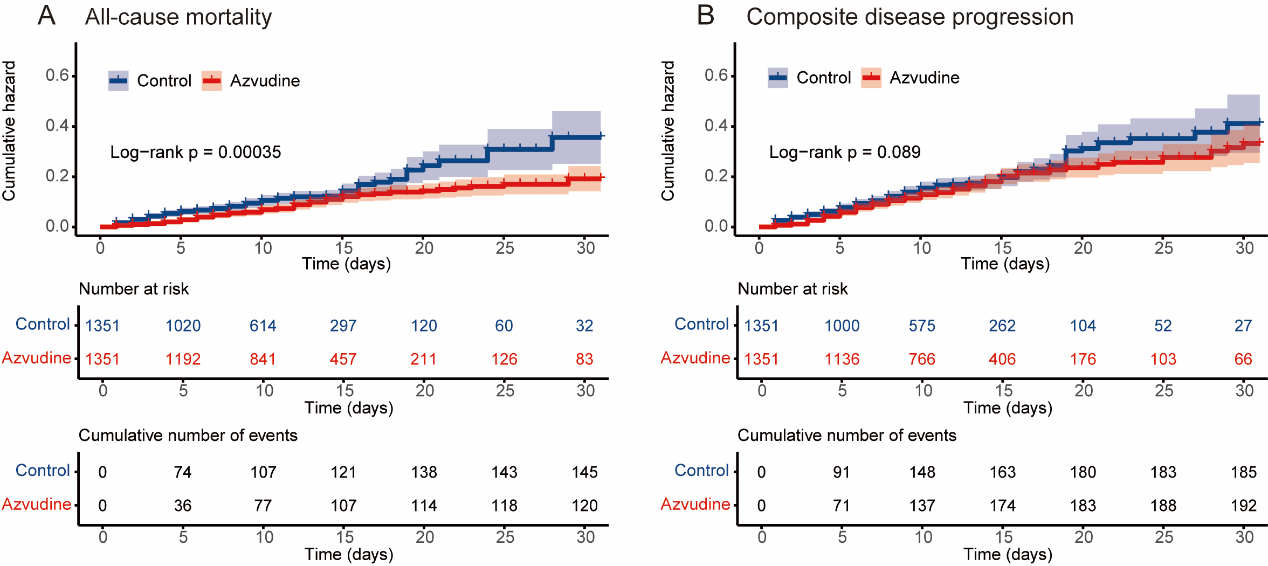


**Figure S6.** **Kaplan–Meier curves of patients receiving Azvudine treatment versus control in the sensitivity analysis where patients who discharged within one day after receiving antiviral treatment were excluded.** Cumulative hazard of all‐cause mortality (A) and composite disease progression (B).


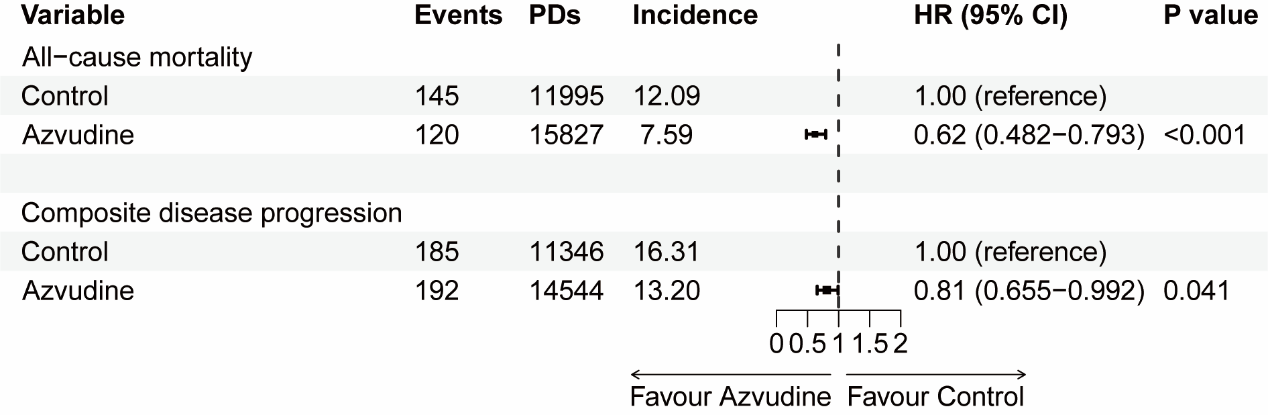


**Figure S7 Multivariate Cox proportional hazards regression analysis of all-cause mortality and composite disease progression in patients receiving normal and Azvudine treatment in the sensitivity analysis where patients who discharged within one day after receiving antiviral treatment were excluded.** Adjusted for all baseline covariates in Table S4. HR: Hazard Ratio; 95% CI: 95% confidence interval. PDs: Person-days. Incidence: events/per 1000 PDs.


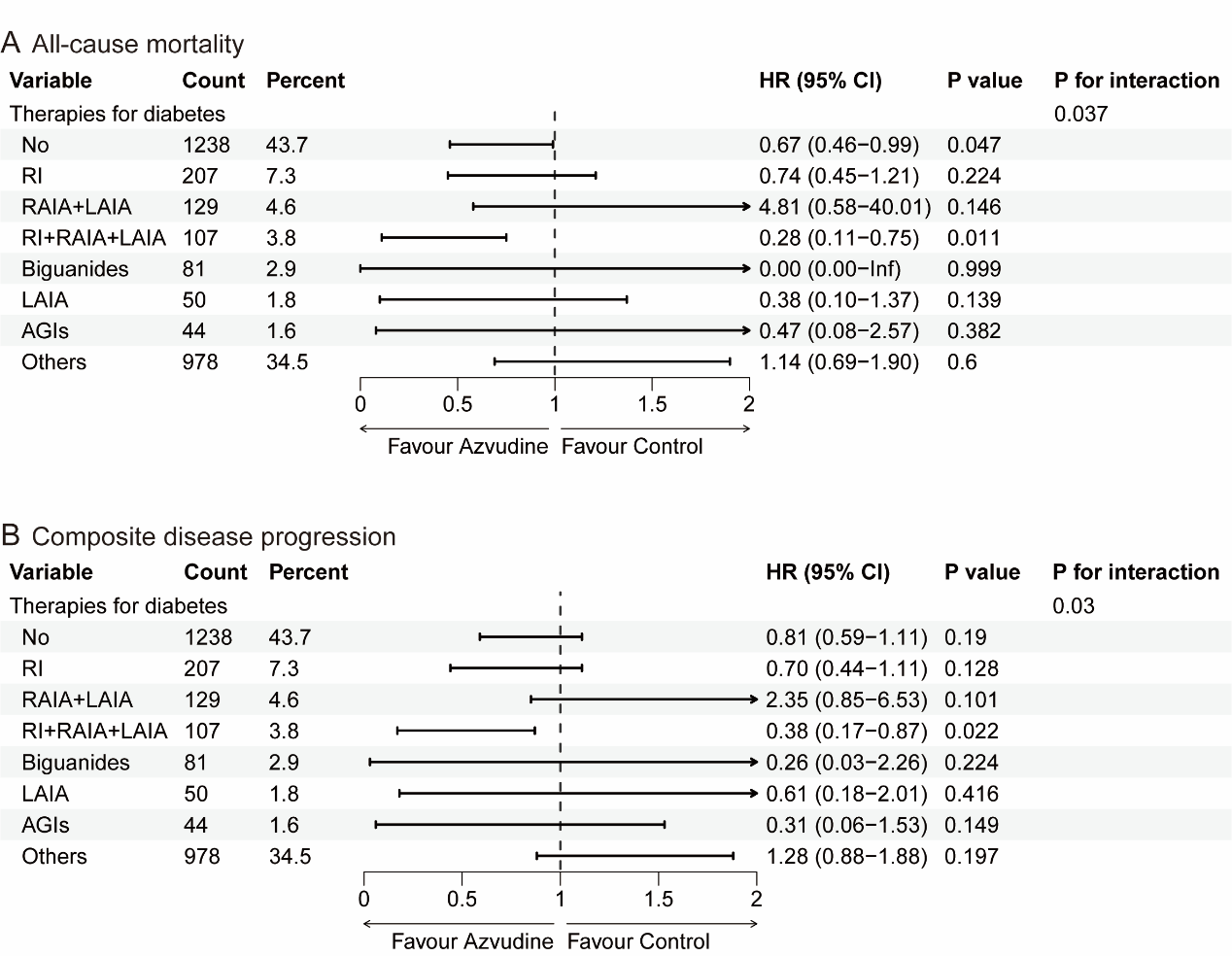


**Figure S8 Subgroup analysis of different therapies for diabetes for the effectiveness of Azvudine in reducing the risk of all‐cause mortality and composite disease progression.** RI, regular insulin; RAIA, rapid-acting insulin analogue; LAIA, long-acting insulin analogue; AGIs, α-glucosidase inhibitor.
